# Synchronous Sigmoid Colon Cancer Seeding a Fistula-in-Ano

**DOI:** 10.7759/cureus.1504

**Published:** 2017-07-23

**Authors:** George E Fowler, Christopher J Young

**Affiliations:** 1 The Medical School, University of Sheffield, United Kingdom; 2 Colorectal Unit, Royal Prince Alfred Hospital

**Keywords:** synchronous sigmoid colon cancer, adenocarcinoma, liver metastases, pan-proctocolectomy, fistula-in-ano

## Abstract

A seeded fistula-in-ano from a synchronous sigmoid colon cancer is rare. The literature is still divided regarding the standard treatment, although an abdominoperineal resection is considered the best option when curative resection is possible.

This case is distinct from previous reports, as the patient had known metastatic liver disease before surgery, and proceeded with a pan-proctocolectomy after neo-adjuvant chemotherapy. The patient died 20 months post-operatively of his metastatic liver disease, having been otherwise asymptomatic for eight months on continued chemotherapy, before commencing palliative treatment (completed five cycles).

Given its rarity, a low suspicion to biopsy a fistula-in-ano is advocated, and the exclusion of malignancy should be considered prior to surgery.

## Introduction

A seeded fistula-in-ano from a synchronous adenocarcinoma of the colon or rectum is rare. Till date, only 28 cases have been reported since the report by Guiss, et al. in 1954 [1-3]. Given its rarity and often late presentation of symptoms (typically attributed to perineal disease), a low suspicion to biopsy a fistula-in-ano has been advocated [4].

It is now widely recognised that exfoliated cancer cells can implant distally on abnormal or damaged mucosa, including wounds and anastomotic or iatrogenic sites; stapled or biopsied [5-6]. The same mechanism is anticipated in the case of a synchronous sigmoid colon cancer and a seeded fistula-in-ano [2]. We report a case which illustrates the potential for exfoliated tumour cells originating from the lumen of the sigmoid colon to seed a fistula-in-ano, a mechanism rarely reported in the literature.

## Case presentation

A 53-year-old man presented with a four-week history of a discharging perianal lump. This had been causing him intermittent discomfort and fresh rectal bleeding. He had a long history of symptoms which had been attributed to haemorrhoids; he was taking irbesartan and atorvastatin tablets for his hypertension and hyperlipidaemia respectively. His sister had Hodgkin’s lymphoma and there was a family history of bowel cancer from his grandmother. He had never smoked but consumed alcohol thrice a week.

On examination, his abdomen was soft and non-tender, while his perineum revealed external haemorrhoids and a hard lump (2 cm in diameter) with two discharging points in the right anterior perianal position (2 cm from the anal verge) which looked concerning. A digital rectal examination revealed what felt like an extra-sphincteric mass. The patient was immediately forewarned of the possibility of a malignant infiltrated fistula tract and a proximal bowel cancer.

Initial investigations included a colonoscopy, examination under anaesthetic, and a staging computed tomography (CT) scan of his abdomen and pelvis, which revealed no liver metastases or lymphadenopathy. On colonoscopy, there were two synchronous sigmoid tumours (4 cm and 5 cm in diameter), which were 25 cm and 30 cm respectively from the anal verge. The proximal tumour prevented progression of the scope. Biopsies of both these tumours revealed infiltrating adenocarcinoma.

A rectal polyp (6 mm in diameter) was found 6 cm from the anal verge, and the previously mentioned right anterior perianal hard lump was 2 cm from the anal verge. The hard lump had two fistula openings. The Lockhart mummery probes demonstrated that these were high right anterior trans-sphincteric fistula-in-ano’s. Histology of the external openings of the fistulas revealed skin with infiltrating adenocarcinoma, while the rectal polyp showed ulceration with acutely inflamed granulation tissue.

The patient was discussed at a multi-disciplinary meeting and subsequently underwent long-course neoadjuvant radiotherapy with 45 Gray (Gy) to the pelvis and perineum, with a 5.4 Gy perineum boost, and chemotherapy with 5-fluorouracil.

**Figure 1:**
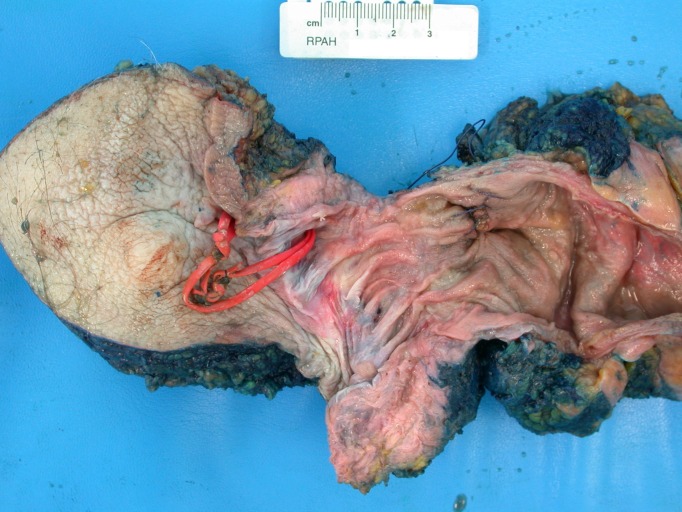
Pan-proctocolectomy specimen Abdominoperineal excision of the rectum and anus component of the pan-proctocolectomy specimen, with extended buttock skin excision, with red vessel-loops in-situ in fistula-in-ano with mucinous adenocarcinoma malignant infiltration.

The patient was planned for a repeat CT scan at six weeks post adjuvant therapy and for surgery four days after. On the day of the CT scan, the patient developed a large bowel obstruction (LBO), but underwent the CT scan which revealed an LBO from the distal sigmoid cancer and multiple 1-2 cm liver metastases. He was admitted that same day and an uncovered stent was placed through the obstructing distal sigmoid cancer. Four days later he underwent a pan-proctocolectomy and end ileostomy formation (Figure 1).

**Figure 2:**
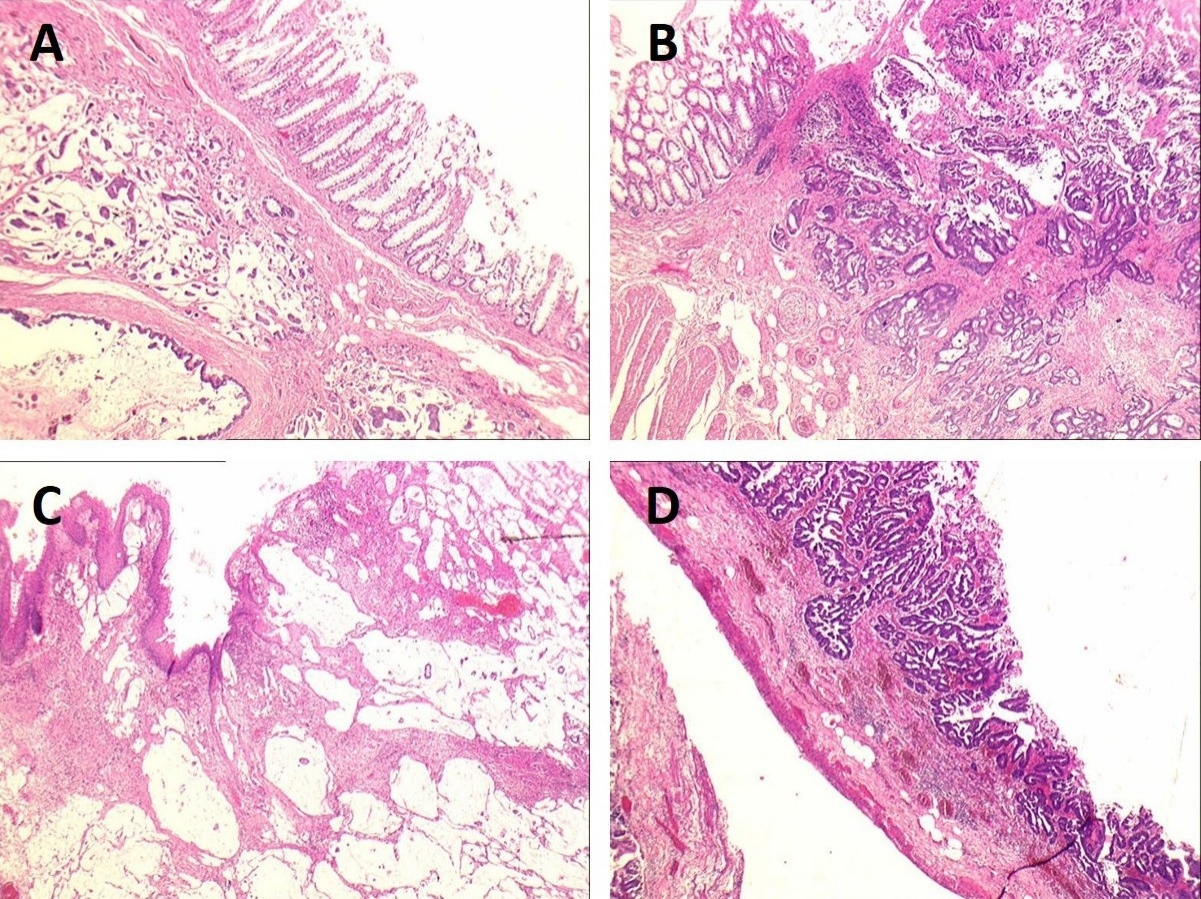
Microscopic views of the sigmoid, fistula-in-ano and caecum (A and B) Synchronous sigmoid mucinous adenocarcinomas; (C) mucinous adenocarcinoma within the fistula tract; (D) the caecum shows a tubulovillous adenocarcinoma with adenocarcinoma in situ (hematoxylin and eosin stain x25).

The histology report revealed two synchronous sigmoid adenocarcinomas with mucinous differentiation and a perianal fistulous tract with invasive mucinous adenocarcinoma (Figures 2A-C). There was metastatic adenocarcinoma in both the peritoneum and 4 out of the 20 lymph nodes (T3N2M1; tumour/node/metastasis (TNM) classification for staging colorectal cancer according to the American Joint Committee on Cancer (AJCC) 2002 TNM system). The caecum had a tubulovillous adenoma with adenocarcinoma in situ and a microscopic focus of invasive adenocarcinoma (T1N0; TNM classification for staging colorectal cancer according to the AJCC 2002 TNM system; seen in Figure 2D). Immunohistochemical studies were not performed. Postoperatively the patient received six cycles of FOLFOX chemotherapy (5-fluorouracil, leucovorin, and oxaliplatin) as a treatment for metastatic bowel cancer with liver metastases.

Post-operatively, a repeat positron emission tomography (PET) scan revealed a ‘mixed response’ to chemotherapy, showing new liver lesions and no extra-hepatic disease. These findings were consistent with a rising carcinoembryonic antigen (CEA) of 1500 µg/L and worsening liver function tests. The patient continued on chemotherapy and was otherwise asymptomatic for eight months. There was initially interval reduction in size and volume of the hepatic metastases. However, the patient’s liver metastatic disease progressed and his treatment changed to folfiri and cetuximab as palliative treatment (completed five cycles). The patient eventually died of his metastatic liver disease 20 months post-operatively.

## Discussion

We describe a case where synchronous sigmoid colon cancer seeded a fistula-in-ano. Although rare, other case reports have also concluded this when implanted cells in the fistula share the same histology as the primary cancer from the sigmoid or rectum [1]. This includes the presence of mucinous material, as noted in our case [4]. Immunohistochemical staining for cytokeratin 7 (CK7) and cytokeratin 20 (CK20) can also be used to support the diagnosis, by distinguishing a colorectal adenocarcinoma from an anal gland carcinoma [2].

A small percentage, 0.1% of all anal fistulae, will however represent a primary cancer resulting from a persistent anal fistula, with recurrent inflammation for at least 10 years and in the absence of a proximal primary adenocarcinoma [1]. These features were not illustrated in this case. In this case, histology revealed a mucinous adenocarcinoma in both the synchronous sigmoid colon cancer and the fistula-in-ano. This demonstrates that exfoliated tumours cells have the potential to seed a fistula-in-ano.

With only 28 reported cases of an adenocarcinoma seeding a fistula-in-ano, there is still a divide in the literature with regards to a standard treatment [1-2]. An abdominoperineal resection (ARP) is considered the best option when curative resection is possible [7]. However, in this case, the patient also had metastatic liver disease, which showed progression while on chemotherapy. We performed a pan-proctocolectomy after neo-adjuvant chemotherapy, because the patient developed an LBO. Three other case reports have also demonstrated lymph node involvement, with metastases in the liver, peritoneum, or lungs [1,7-8]. However, as there are a limited number of reports, with short periods of follow-up, the prognosis and best therapy for individual patients cannot be determined.

## Conclusions

This case highlights the rare nature of a synchronous sigmoid colon cancer seeding a fistula-in-ano. The best surgical approach remains controversial. Nevertheless, a low suspicion to biopsy a fistula-in-ano is still advocated, as should the exclusion of proximal colorectal pathology, including malignancy.
